# Strengthening excellence in agricultural sciences education through mentorship systems for undergraduate students to promote sustainable agricultural development

**DOI:** 10.1371/journal.pone.0332144

**Published:** 2025-09-11

**Authors:** Shenggang Pan, Zhaowen Mo

**Affiliations:** College of Agriculture, South China Agricultural University, Guangzhou, China; Instituto Tecnologico Autonomo de Mexico, MEXICO

## Abstract

The study examines the role of mentorship in delivering high-quality undergraduate education in the agricultural sciences, and to find ways to improve mentorship systems in agricultural science education and promote sustainable agricultural development. A questionnaire survey was conducted with 329 samples, as well as a case study on a mentorship system. The questionnaire comprised 7 topics and 20 items related to mentoring relationships. The results show that mentorship played a crucial role in cultivating excellent students in agricultural science by exerting significant influences on their experience/skills/technical abilities, innovation and practical abilities, and team-work spirit. Decision-making and problem-solving abilities were also noticed as well. For mentorship opportunities to be successful, students must be aware of them. They should be of excellent quality and receive support from national bodies. Agricultural sciences education should help students fully understand the mentorship systems. Our findings offer both theoretical significance and practical value for the establishment of innovative approaches for nurturing agricultural talent and for promoting the sustainable agricultural development.

## Introduction

The ongoing growth of the global population is fueling increased demand for food and a wide range of other agricultural products. This has raised a plethora of challenges related to agriculture that require profound changes to agricultural practices worldwide. Skilled agricultural scientists and managers must now meet the increased demand for food and bio-industrial products [1,2].

Agricultural education plays a key role in spearheading transformative changes in agriculture [3], and it is being continually refined to provide more chances for students to adapt to the demands of modern agriculture [4,5]. If agricultural education can effectively integrate advanced technologies into the curriculum, this will hone students into skilled agricultural professionals and propel modern agricultural practices into the future [6]. For example, Tian et al. [7] found that agricultural technical education has a notable capacity to substantially mitigate pesticide expenditure by farmers engaged in vegetable cultivation. However, the issue of students graduating with insufficient skills remains a pressing concern in contemporary agricultural education.

Higher education programming in agricultural studies requires significant resources to facilitate opportunities for the practical application of knowledge. Mulder and Pachuau reported that students in agricultural fields were expected to acquire knowledge beyond primary production and the traditional agricultural domains [8]. The mentorship system is crucial for enhancing students’ education by fostering the acquisition of knowledge and skills [9–11]. Its effectiveness can be influenced by factors such as power dynamics, gender, age, and cultural differences [12]. For instance, one study found that faculty mentorship played a vital role in providing undergraduate students with more opportunities to develop their research skills during a Summer Undergraduate Research Experience [13]. Therefore, in undergraduate agriculture programs, mentorship has the potential to improve students’ understanding of agricultural science, equip them with practical skills, and stimulate research and innovation under the guidance of experienced mentors. This, in turn, contributes to the continuous advancement of agricultural education.

## Undergraduate mentorship in agricultural education

In undergraduate mentorship, the mentor imparts knowledge to students and helps to improve students’ research and problem-solving abilities, and their spirit of teamwork. Undergraduate mentorship must also consider students’ mental health and guide students to make appropriate career plans. Mentorship is distinguished from other interpersonal relationships by the depth of support and personalized interaction between the mentor and the mentee [14]. Many universities in China have reintroduced the undergraduate mentorship system [15]. The Ministry of Education of China has released reports on the strengthening of undergraduate education in higher education, and has expressed growing government interest in advancing the undergraduate mentorship system. The reason for this is the mentorship system’s potential for nurturing talent, improving education, driving reforms, and developing skills. A mentorship system adapted to the demands of today’s higher education and the various professions is vital for education and talent cultivation in China.

In China, food security is a top priority for ensuring economic and social stability. Food security is dependent on modern technology, comprehensive agricultural education, and advanced agricultural research [16,17]. On September 19th, 2022, the Ministry of Education of China issued guidelines to advance new talent development in agricultural science and technology, focusing on modernizing agricultural education, cultivating leaders in rural modern agriculture, and implementing initiatives to benefit both farmers and the environment. The guidelines also addressed the importance of mentorship for students in agricultural fields. The mentorship system operates as a form of experiential learning, in which mentors guide undergraduate students and prepare them for the field [18–20]. Mentorship is crucial for teaching students how to apply agricultural theory and technology to realistic scenarios, with mentors identifying and teaching the essential knowledge, skills, behaviors, and attitudes required of future food production professionals [21,22]. Despite significant advancements in agricultural education, there has been a striking absence of research on the importance of mentorship systems for agricultural students, which poses a significant barrier to improving student success in agricultural science programs. A deeper understanding of students’ perspectives on mentorship in agricultural colleges could enhance the quality of educational offerings in this field [23].

As it is not yet clear whether mentorship systems can promote the quality of undergraduate talents in agricultural science, the present study mainly addresses the influence of mentorship systems on undergraduate’ experience/skills/technical abilities, problem-solving ability, teamwork spirit, innovation ability, and mental health by conducting a questionnaire and case study on a mentorship system. This research is vital for substantiating the important ability of undergraduate mentorship to enhance high-quality agricultural sciences education in China. Its findings offer both theoretical significance and practical value in the establishment of innovative approaches for nurturing agricultural talent.

## Methodology

This study was conducted at South China Agricultural University (SCAU), which was established in 1909 and enjoys a distinguished reputation in its field. According to the policies of the College of Agriculture in SCAU, every teacher with a doctoral degree or an associate senior title can act as mentors, and all undergraduates majoring in agricultural science can be mentees. Feedback was collected from undergraduate students in the College of Agriculture in SCAU to capture their perspectives on the mentorship system. Questionnaires and a case study were used in this research. While the questionnaire measured students’ viewpoints on the skills offered by mentorship programs, the case study was designed to explore the implications of a particular mentorship program in the Rice Research Laboratory, College of Agriculture, SCAU. The quantitative method was used in the current study.

### Questionnaire

A written questionnaire was developed to better understand the influence of mentorship systems on students’ professionalism, and on their technical/innovation-related, communication, leadership, self-management, decision-making, and problem-solving skills. These competencies were categorized into seven topics, and students assessed the impact of mentorship on these skills through a set of 20 items (Table 1). The questionnaire was established according to the needs of both agricultural employers and agricultural students, along with insights into agricultural higher education from teachers in the field of agricultural science, as reported in the study by Parrella et al. [24]. There were 20 items in the questionnaire and seven topics, as recommended by Haselberger et al. [11] (see Table 1). Quantitative data were collected through this method as well.

**Table 1:** Topic and items in the questionnaire on the impacts of mentorship in agricultural studies.

Topic	Item
(1) Experience/ skills/ technical abilities	
	The impact of mentorship on students’ innovation ability
	The impact of mentorship programs on students’ practical abilities
	The impact of mentorship programs on students’ employment competitiveness
(2) Teamwork	
	Opportunities to participate in a mentor’s research projects
	Opportunities to conduct innovative projects and publish papers under the guidance of a mentor
(3) Communication	
	Students’ understanding of their role and responsibilities in the mentorship system
	The effectiveness of communication with the mentor
	The positive relationship with the mentor
(4) Leadership	
	The influence of the mentorship system on students’ leadership abilities
(5) Decision/ problem solving	
	The impact of the mentorship system on students’ career planning and employability
	The influence of the mentorship system on students’ academic skills and research proficiency
	The impact of the mentorship system on students’ ability to overcome difficulties and solve problems
(6) Self- management	
	The impact of the mentorship system on students’ sense of social responsibility
	The impact of the mentorship system on students’ social adaptation ability
	The impact of the mentorship system on fostering students’ autonomy and independence
	The impact of the mentorship system on students’ personal growth
	The impact of the mentorship system on students’ mental health
(7) Professional spirit	
	The impact of the mentorship system on students’ understanding of modern agricultural development frontiers and trends
	The impact of the mentorship system on students’ international perspectives on agricultural science and technology
	The impact of the mentorship system on students’ awareness and enthusiasm for innovation.

### Study sample

A valid sample of 329 agricultural students from South China Agricultural University participated in this study. They represent students from a range of academic levels. Questionnaires with all items completed were identified as the valid questionnaires. The data collected were subsequently analyzed to assess the participants’ views on the mentorship system. Variables such as academic year and gender were considered in the analysis. Of the participants, 144 (43.8%) were male and 185 (56.2%) were female. Furthermore, 18.5% were in their first year (freshmen), 29.8% were in their second year (sophomores), 30.1% were in their third year (juniors), and 21.6% were in their fourth year of undergraduate studies (seniors). To explore the implications of the mentorship system, the case study analyzes the mentorship system at the College of Agriculture, South China Agricultural University, and its application at the Rice Research Laboratory.

### Administration of the questionnaire and data analysis

The questionnaire was first forwarded online to every class teacher in the College of Agriculture, who then forwarded it to all undergraduates in there classes. Stringent measures were implemented to prevent communication between students while they completed the survey. The Statistical Package for Social Sciences (SPSS) software was used for data analysis.

## Results

### Undergraduate students’ viewpoints on mentorship in agricultural colleges

**Fig 1:**
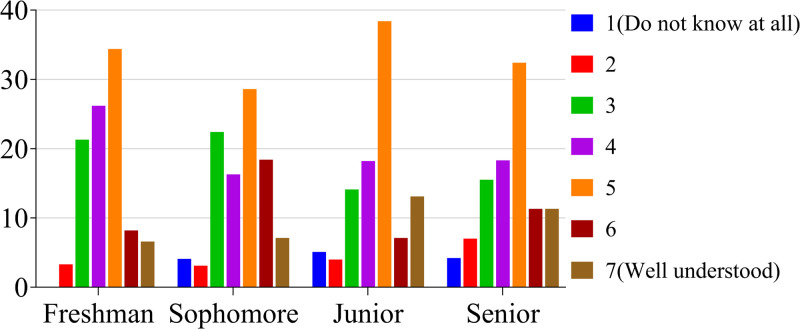
Proportion of students in each grade with varying levels of knowledge about the mentorship system (%).

According to the method of Paralle et al., undergraduate students’ familiarity with the mentoring system was quantified on a seven-point scale of 1 to y, with from 1 = completely unknown to 7 = very understood [24]. The results showed that most students had a basic grasp of the mentorship system, and that the senior students were more familiar with the mentorship system than the freshman and sophomore students (Fig 1).

**Fig 2:**
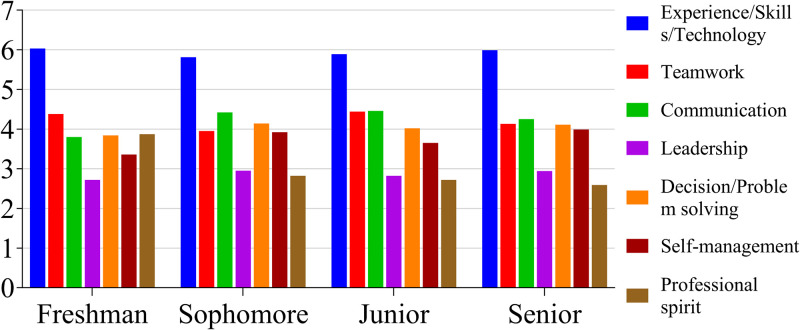
Mean perception of abilities among students in each grade (*N* = 329).

Based on the undergraduate agricultural students’ understanding of the mentorship system, a survey was conducted of their self-assessment of the seven abilities enumerated above. As the results presented in Fig 2 indicate, the students generally considered experience/skills/technical abilities to be very important; however, across all grades, the students did not place high value on leadership. Students in their second, third, and fourth years also did not consider professional spirit to be significant. The importance of teamwork, communication, decision-making/problem-solving, and self-management were assessed as having a medium level of significance.

**Fig 3:**
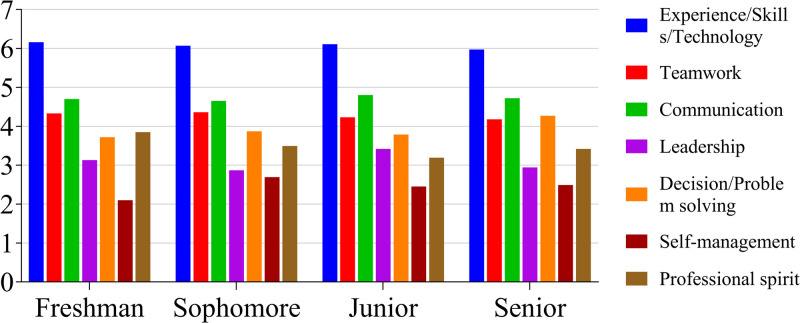
Mean values of students’ perceptions of the impact of the mentoring system on the development of abilities, divided by grade (*N* = 329).

The results show that the students generally believed that the mentorship system had a significant impact on their experience/skills/technical abilities. Students across all grade levels reported that its influence on their self-management skills was not very important, and that is was even less crucial to the nurturing of leadership skills. The importance that the students ascribed to the mentorship system in terms of cultivating their teamwork, communication, and decision-making/problem-solving skills, as well as their professional spirit, was moderate (Fig 3).

**Fig 4:**
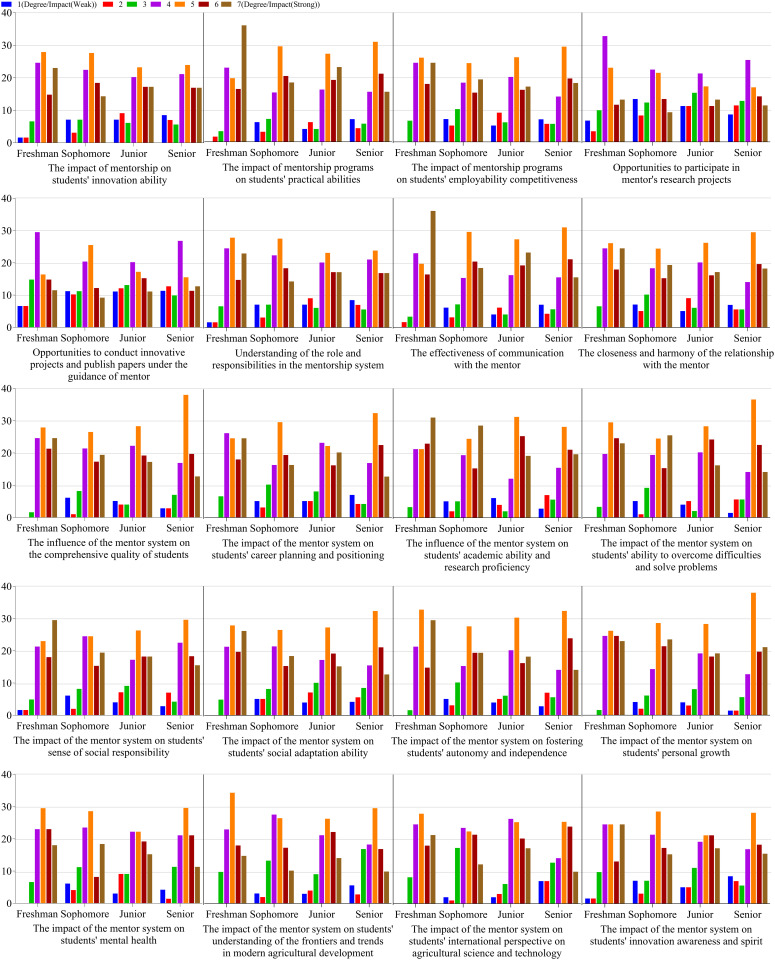
The percentage of students across grades with different perceptions of the impact of the mentorship system on their abilities (%).

Further analysis of the data revealed that the students felt that the mentorship system had a significant impact on their innovation and practical abilities, as well as their employment competitiveness. The mentorship system provides students with opportunities to participate in research projects, carry out innovative research projects, and write publications. Overall, the students were aware of their roles and responsibilities in the mentorship system, and they reported having a close relationship and good communication with their mentors. Although the students considered leadership skills to be less important, they reported that the impact of the mentorship system on leadership skills was significant. Most students also acknowledged the importance of the mentorship system in developing their decision-making and problem-solving skills, and especially in improving their career prospects and employability, academic competencies and research proficiency, and their ability to overcome difficulties. They reported that the mentorship system also had a significant impact on their sense of social responsibility, social adaptability, autonomy and independence, personal growth, psychological health, students’ understanding of modern agricultural development frontiers and trends, international perspectives on agricultural technology, and their awareness and enthusiasm for innovation (Fig 4 and Table 2).

**Table 2:** The mean value of the students’ perceptions across different grades of the impact of the mentorship system on their competencies (*N* = 329).

Abilities	Freshman	Sophomore	Junior	Senior
	(n = 61)	(n = 98)	(n = 99)	(n = 71)
The impact of mentorship on students’ innovation ability	5.11(1.42)	4.72(1.62)	4.64(1.78)	4.63(1.78)
The impact of mentorship programs on students’ practical abilities	5.54(1.36)	4.94(1.63)	5.07(1.63)	4.85(1.65)
The impact of mentorship programs on students’ employment competitiveness	5.30(1.27)	4.71(1.75)	4.71(1.69)	4.86(1.72)
Opportunities to participate in a mentor’s research projects	4.49(1.58)	4.07(1.80)	4.08(1.85)	4.18(1.76)
Opportunities to conduct innovative projects and publish papers under the guidance of a mentor	4.33(1.65)	4.12(1.77)	4.10(1.85)	4.07(1.85)
Students’ understanding of their role and responsibilities in the mentorship system	4.66(1.25)	4.41(1.48)	4.52(1.57)	4.49(1.47)
The effectiveness of communication with the mentor	4.98(1.42)	4.88(1.61)	4.88(1.49)	4.89(1.55)
The positive relationship with the mentor	5.20(1.24)	5.23(1.44)	5.20(1.38)	5.17(1.52)
The influence of the mentorship system on students’ leadership abilities	5.43(1.16)	4.91(1.61)	4.91(1.57)	4.94(1.37)
The impact of the mentorship system on students’ career planning and employability	5.28(1.28)	4.86(1.59)	4.82(1.67)	4.82(1.61)
The influence of the mentorship system on students’ academic skills and research proficiency	5.57(1.23)	5.16(1.64)	5.11(1.60)	5.01(1.58)
The impact of the mentorship system on students’ ability to overcome difficulties and solve problems	5.44(1.15)	5.05(1.63)	5.01(1.52)	5.03(1.38)
The impact of the mentorship system on students’ sense of social responsibility	5.34(1.45)	4.83(1.63)	4.82(1.65)	4.86(1.51)
The impact of the mentorship system on students’ social adaptation ability	5.41(1.23)	4.79(1.64)	4.75(1.62)	4.80(1.55)
The impact of the mentorship system on fostering students’ autonomy and independence	5.49(1.18)	4.93(1.63)	4.89(1.56)	4.94(1.51)
The impact of the mentorship system on students’ personal growth	5.43(1.15)	5.19(1.54)	4.96(1.55)	5.28(1.30)
The impact of the mentorship system on students’ mental health	5.23(1.19)	4.62(1.65)	4.70(1.62)	4.79(1.45)
The impact of the mentorship system on students’ understanding of modern agricultural development frontiers and trends	5.05(1.19)	4.65(1.40)	4.87(1.49)	4.54(1.55)
The impact of the mentorship system on students’ international perspectives on agricultural science and technology	5.20(1.26)	4.77(1.42)	4.99(1.42)	4.55(1.70)
The impact of the mentorship system on students’ awareness and enthusiasm for innovation	5.07(1.48)	4.74(1.63)	4.79(1.67)	4.66(1.76)

Note: Mean (standard deviation).

**Fig 5:**
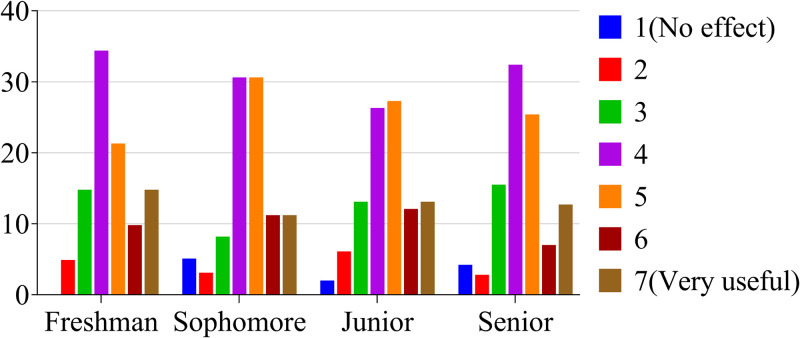
Proportion of students from different grades based on their recognition of the value of their role in rural revitalization (%).

Students were asked about their role in providing services for contemporary rural revitalization, and the results show that their attitudes overall were not very positive (Fig 5).

### Qualitative data on student perspectives on mentorship

The mentor’s level of involvement significantly influenced most students’ perception of their relationship. For example, when describing their understanding of their role and responsibilities in the mentorship system, one respondent shared the following:

*Yes, I understand the concept of mentorship. In the context of China, the mentorship system often involves a more experienced individual guiding and advising a less experienced person, especially in educational or professional settings. It plays a crucial role in Chinese culture and education, fostering relationships between students and teachers, as well as in various industries where experienced professionals mentor newcomers to help them succeed and grow.*

This respondent further elaborated on how mentorship affected how they understand frontiers and trends in modern agricultural development:

*The mentorship system has a significant impact on my understanding of the forefront and trends in modern agriculture in China. By fostering close relationships with mentors, individuals, particularly students and newcomers in the agricultural field, can gain valuable practical experience and specialized knowledge. Mentors often possess extensive industry experience and can share insights into new technologies, sustainable agricultural practices, and market trends. This knowledge transfer helps the new generation of practitioners in the agricultural sector better adapt to the challenges and opportunities of modern agriculture, promoting innovation and sustainable development within the industry. Therefore, the mentorship system greatly enhances my understanding of the forefront and trends in modern agriculture, especially within the Chinese context.*

They further descirbed the importance of effective communication between mentor and mentee:

*Yes, I have maintained highly effective communication with my mentor. This communication has been instrumental in many ways, including learning experimental techniques, as well as writing and revising research papers. We have had open and constructive discussions, allowing me to grasp new laboratory methods and refine my research writing skills, ensuring that I receive valuable guidance and feedback throughout the process.*

The respondent also noted how mentorship provided them with opportunities to participate in their mentor’s research projects:

*I have had numerous opportunities to participate in my mentor’s research projects. Most notably, I’ve had the chance to lead my own graduate thesis project, and I’ve also been involved in various aspects of my mentor’s larger research initiatives. These opportunities have allowed me to gain hands-on experience and contribute meaningfully to ongoing research endeavors under my mentor’s guidance.*

The respondent noted the influence of the mentorship system on the comprehensive quality of students:

*The mentorship system has had a profound impact on my overall qualities and abilities. My mentor has not only imparted practical laboratory skills but also taught me how to design experiments for research projects. This mentorship has significantly enhanced my research capabilities and has contributed to my well-rounded development.*

Lastly, they described the overall impact of mentorship:

*The mentorship system has had an extremely positive impact on me, especially as a student who was initially inexperienced in research. Through close collaboration with my mentor, I not only acquired essential practical skills and knowledge but also learned how to formulate and address research questions, design experiments, and write research papers. My mentor’s guidance and support helped me establish a solid research foundation and provided invaluable insights and opportunities for my career development. In summary, the mentorship system has played a crucial role in shaping my academic and professional path, and I am highly satisfied with its influence on me.*

## Case study on a mentorship system

The agricultural science program at the College of Agriculture in South China Agricultural University is nationally recognized as a site where a “First-Class Discipline” is taught. It is also acknowledged as a national-level distinctive specialty, serving as a pilot program for nurturing exceptional agricultural and forestry talent at the national level. Furthermore, the program stands out as a key, pivotal specialty with unique attributes in Guangdong Province. The goal of the agricultural science program is to nurture well-rounded graduates who possess a solid foundation in the fundamental theories, knowledge expertise, and practical skills related to crop production, crop breeding, and the production of high-quality seeds. Since its establishment, the agricultural science program has been taught through teachers and thesis mentors.

To enhance the development of exceptional undergraduate talent, in 2012 the College of Agriculture established the Ding Ying Innovation Class, a pilot undergraduate mentoring initiative. All students in the Ding Ying Innovation Class can choose any teacher from the SCAU College of Agriculture as their advisors, according to their career plans. With the consent of their advisors, the students can finish their practical training, and their thesis research and writing under the guidance of their advisor. During the pilot phase, the positive effects of this undergraduate mentoring system became apparent. Undergraduate education is the foundation of talent training in the College of Agriculture and thus the training of proficient undergraduate students is prioritized there. In 2017, the College of Agriculture proposed the implementation of an undergraduate mentoring system at South China Agricultural University. The goals of this initiative were to harness the influential role of teachers in talent cultivation, establish a fresh teacher-student dynamic, implement high-quality education and training, elevate students’ competence and their innovative mindset, and ultimately enhance the standard of college education and the level of agricultural science talent, in line with society’s needs for high-quality undergraduate education. The implementation of such a system in agricultural science is significant for enhancing students’ overall abilities and spirits of innovation, revamping teacher-student relationships, implementing quality education and personalized training, improving education offerings at the institution, and producing high-quality talent in the field of agricultural science.

**Fig 6:**
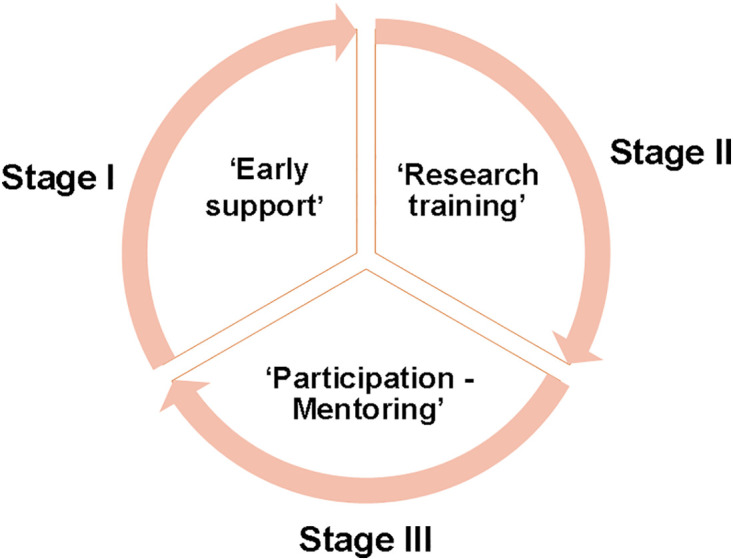
The framework for cultivating undergraduate students in agricultural studies at the Rice Research institute of SCAU.

To better educate undergraduate students in agricultural studies and to foster innovative talent, the College of Agriculture has improved and enriched its mentorship system with tangible measures such as creating an “ Early support, Research training, Participation - Mentoring” framework for cultivating undergraduate students in agricultural studies at the Rice Research Institute of SCAU (Fig 6).

(1) ***‘Early support’:*** In this stage, students first enter the laboratory and are initiated into scientific research training, and are expected to make timely submissions of research suggestions.This stage is mainly designed to allow students to become familiar with laboratory work and develop experimental skills early in their academic journey. Students are also encouraged to present their ideas during the course of their experiments and discuss them with their mentors, thus helping them in the development of their scientific and independent thinking skills. Scientific research training at this stage can also contribute to honing the students’ problem-solving skills.
(2) ***Research training:*** This stage encompasses the completion of undergraduate thesis research and innovative training. Students must write their undergraduate thesis and undertake comprehensive research training. Through these research activities, the students gain a deeper understanding of research methodologies and the skills required to conduct scientific research, thereby establishing a solid scientific research foundation. Moreover, in this stage, the students participate in systematic academic training and scientific and technological competitions. These competitions provide the students with opportunities to demonstrate their abilities alongside their peers, improve the overall quality of their work, hone their practical skills and awareness of innovation, and cultivate their team spirit.
(3) ***Participation – Mentoring:*** In this stage, students from upper grades mentor undergraduate students in earlies grades, thereby transferring their laboratory, technology-related, and basic experimental skills to them. The act of passing on their experimental abilities and scientific research competencies to junior students contributes to their personal growth and strengthens the connections and communication among peers in different grades. Overall, this leads to a more positive academic atmosphere and fosters team spirit.

Through this model, an analysis of statistics from data 2020–2023 on undergraduate students in the mentorship program was conducted, revealing that a total of 19 students actively participated in 11 scientific and technological innovation projects, and eventually graduated. They won 12 awards and honors at or above the university level, including the “Challenge Cup” at the Guangdong Provincial College Students Extracurricular Academic Science and Technology Works Competition, and the Guangdong Province Undergraduate Plant Production Major Graduation Thesis Competition. The findings show that 17 papers were published with students in the program listed as the first author, including 16 SCI indexed papers, with a cumulative impact factor of 56.465. This indicates that the tutoring system has played an important role in promoting students’ involvement in academic research and preparing them for advanced studies at superior institutions. Of the undergraduates who graduated during the time under study, 83.81% went on to enroll in postgraduate education at universities such as Peking University, Zhejiang University, the University of the Chinese Academy of Sciences, China Agricultural University, Shanghai Jiao Tong University, Southern University of Science and Technology, and South China Agricultural University.

## Discussion

Agricultural science and technology are advancing rapidly, and thus agricultural education is fundamental to developing expertise and the practical applications of that field [25]. To prepare students for careers in academia and industry, a well-rounded education that nurtures both theoretical and practical skills, and provides students with experiential learning opportunities is essential [26]. The mentors at an agricultural college may have different experiences to mentor the agricultural students. Therefore, it is very important to build up a simple training model to which the students can adapt and from which they can develop. Here, we present a mentorship systems framework for cultivating undergraduate students in agricultural studies at the Rice Research Institute of SCAU, which has made excellent efforts to develop the students’ potential. To further understand why student achieved different results from the mentoship system, this research builds upon the insights reported by agricultural students, as reported in the study by Parrella et al. [24].

Notably, it is important that the college and mentor be made aware of students’ concerns. In this study, students were inclined to prioritize experience, skills, and technical abilities as being the most important to them, while leadership was less valued across all grade levels. Second-, third-, and fourth-year students found professional spirit to be significant. Teamwork, communication, decision-making/problem-solving, and self-management were considered moderately important competencies (Fig 2). The different prospects were compared to the results of studies by Robinson and Bryan [27], and Abbey et al., [28] due to the differences in national policies, culture, and the current education and agricultural development context. The main reason may be that they foresaw few opportunities for leadership in their future jobs. The mentorship system was seen as having played a moderate role in fostering students’ teamwork, communication skills, decision-making/problem-solving abilities, and professional spirit (Fig 3). The mentorship system was thus recognized as vital for the development of certain relevant abilities (Fig 4, Table 2).

The attitudes of agricultural students toward their field are of significant importance, as noted by Yueh et al. [23]. Faulkner et al. [29] emphasized the necessity of cultivating students’ interest in agriculture to mitigate negative attitudes toward the field. There is indeed a research gap in understanding the impact of mentorship on students’ interest in agriculture. However, it is evident that students at different academic levels may vary in this regard, potentially because agriculture students in higher grades have had the opportunity, under the mentorship system, to nurture their interest in agriculture during their coursework, scientific research training, thesis research, and scientific and technological competitions, under the mentorship system. Indeed, as emphasized by Mulder and Pachuau [8] and Cosby et al. [30], students in agricultural fields were now expected to possess knowledge that transcends the boundaries of primary production and the traditional agricultural domains. This shift might explain why, when, students’ self-awareness and their involvement in modern rural revitalization initiatives were evaluated, their overall attitude of students did not seem particularly positive. This is probably due to their diminished confidence in their knowledge, technological proficiency, and practical skills (Fig 5). Additionally, this research paper reviewed the mentorship system model within the Rice Research Institute of SCAU and found that it enhanced students’ innovation capabilities, resulting in notable achievements in agricultural studies. This finding agrees with results of a study by Salinitri [31], which found that students who actively engaged in mentorship systems experienced greater academic success. Moreover, it is important not to neglect the effect of the experience of the mentors. They can provide practical skills and stimulate research and innovation experiences. Though the views of tutors on the mentorship system were not investigated in this study. By inquiring about the perspective of the students, the mentors can get a more in-depth understanding of how to more effectively implement the mentorship system.

## Conclusion and implications

Undergraduate agricultural majors are distinct group because they require a broad knowledge base spanning various disciplines. This is necessary because of the complex nature of agriculture, which remains one of humanity’s oldest and most vital production activities. To excel, students must acquire expertise in crop growth, ecology, soil science, genetics, and various agricultural technologies. This complexity requires comprehensive education, highlighting the importance of mentorship programs for undergraduate students.

Mentorship systems for undergraduate students in agriculture are crucial for the development and application of modern agricultural techniques and for fostering new agricultural innovation talents. Students who are not part of such programs face challenges such as a lack of exposure to innovative practices during training and difficulties adapting to modern agricultural trends, despite the high demand for agricultural professionals with these skills across the country. The implementation of mentorship programs in agricultural education is thus imperative for fostering a new generation of agricultural experts. Doing so is beneficial for providing students with access to a mentor’s experience, knowledge, skills, guidance, and counseling, as they can become aware of new innovative practices and develop new abilities. The mentorship training model meets the needs of modern agricultural development and education, while meeting the expectations of undergraduate students who seek to enhance their innovation-oriented training and engage in hands-on learning experiences. This study’s findings on mentorship programs point to some effective measures that can be adopted for institutions, mentors, and students. The institutions are encouraged to review the recommendations in Table 3, which will help them implement strong programs to support students in becoming the experts needed for the future of agriculture. Ideally, the mentors should take the students’ interests into account and give some relevant suggestions on the key issues that have been voiced by different undergraduate students. Every student should attach great importance to the effect of the mentorship system, actively cooperate with the guidance of the mentor, and strive for success. Moreover, the effects of factors such as the gender, age and personality of mentors on the effectiveness of mentorship systems for undergraduate students in agricultural science were not examined in the present study. Future studies could investigate this issue.

**Table 3:** Recommended measures for the evaluation of mentorship programs in agricultural colleges.

Measures	Description
Integrating disciplinary learning with practical experience	*Students should engage in agricultural experiments on campus, to grasp the present and future landscape of agricultural technology. Off-campus visits to farms and agricultural exhibitions offer insights into real-world agricultural production. Practical experience enables students to deepen their understanding of agricultural concepts, apply theory to practice, and develop problem-solving skills.*
Mastering agricultural research skills	*Students must acquire effective oral and written communication skills, especially when engaging with agricultural professionals. Additionally, they need to develop laboratory experimental skills and proficiency in analytical methods to design and execute experiments successfully. Furthermore, the ability to read, assess, and synthesize literature is essential for extracting valuable insights from diverse research sources.*
Achieving goal of cultivating abilities in agricultural technological innovation	*Students need to be able to find innovative solutions to important scientific problems. Generating new ideas and methods is the goal of high-quality undergraduate education in agronomy. Schools can gradually develop students’ ability to choose to answer important scientific questions by conducting science and technology competitions and research projects. Schools should offer innovative and contemporary courses, and encourage students to participate in competitions on science and technology innovation and practical projects. These opportunities would enable students to learn basic methods and techniques for scientific research, develop critical thinking skills, and seek novel problem-solving methods.*
Emphasizing the development of students’ practical skills	*By organizing teaching practicums and internships, students can conduct scientific research and complete hands-on activities in applied agricultural contexts, nurturing their practical and technological innovation abilities. Furthermore, students need practice to better grasp the basic methods and techniques of scientific research, and to convert theoretical knowledge into practical frameworks.*
Focusing on students’ teamwork and leadership abilities	*In modern agricultural education, teamwork and leadership abilities are becoming increasingly important. Schools can organize project practice, competitions, etc., to provide opportunities for students to cooperate and complete tasks as a team, developing their teamwork and leadership abilities. At the same time, schools can also use the mentorship system to teach students how to lead, manage teams and maximize the output from team members.*
Emphasizing the development of students’ overall abilities	*Students also need to have broad knowledge and good humanistic qualities. Schools can cultivate students’ comprehensive qualities by offering diversified courses. Meanwhile, schools can also organize research and production promotion in agriculture, volunteer activities, etc., to expose students to various contexts and cultivate their sense of social responsibility and civic awareness.*

Overall, the mentorship system for undergraduate students majoring in agriculture is a crucial teaching and management model to enhance the quality of talent cultivation in the field of agriculture, through which full-time teachers provide personalized guidance on students’ ideology, academic performance and technical development. This study confirms that mentorship systems are important for strengthening excellence in agricultural sciences education, presents the students’ perspective on a mentorship systems, and provides measures for the development of mentorship systems in agricultural education.
